# PTPRS is a novel marker for early tau pathology and synaptic integrity in Alzheimer’s disease

**DOI:** 10.1002/alz.086435

**Published:** 2025-01-09

**Authors:** Alexandre Poirier, Cynthia Picard, Anne Labonté, Isabelle Aubry, Daniel L. Auld, Henrik Zetterberg, Kaj Blennow, Michel L. Tremblay, Judes Poirier

**Affiliations:** ^1^ McGill university, Montreal, QC Canada; ^2^ Douglas Institute, Montreal, QC Canada; ^3^ Douglas Mental Health University Institute, Montréal, QC Canada; ^4^ University of Gothenburg, Mölndal, Gothenburg Sweden; ^5^ Department of Psychiatry and Neurochemistry, Institute of Neuroscience and Physiology, The Sahlgrenska Academy at the University of Gothenburg, Mölndal Sweden; ^6^ Clinical Neurochemistry Laboratory Sahlgrenska University Hospital, Mölndal Sweden; ^7^ University of Gothenburg, Mölndal Sweden; ^8^ Douglas Mental Health Research Centre, Montreal, QC Canada

## Abstract

**Background:**

Synaptic dysfunction is a central pathologic feature of Alzheimer’s disease (AD), with synaptic loss even preceding neuronal loss in specific brain regions. In healthy individuals, synaptic function and plasticity are orchestrated through the complex integration of signaling inputs generated by cell surface receptors.

**Methods:**

In this study, we investigate the role of one such receptor, protein tyrosine phosphatase receptor sigma (PTPRS), in the context of Alzheimer’s disease. Publicly available datasets (BRAINEAC, ROSMAP, ADC1) and a cohort of asymptomatic but “at risk” individuals (PREVENT‐AD) were used to explore the relationship between PTPRS and various Alzheimer’s disease biomarkers.

**Results:**

We identified that PTPRS rs10415488 variant C shows features of neuroprotection against early phospho (181)‐tau pathology (p<0.005) and synaptic degeneration (GAP‐43, p<0.001) in Alzheimer’s disease. In brain tissues, PTPRS protein abundance was significantly correlated with the quantity of two markers of synaptic integrity: SNAP25(R^2^=0.20, p < 0.0001) and SYT‐1(R^2^=0.12, p < 0.0001). We also found the presence of sexual dimorphism for PTPRS, with higher CSF concentrations in males (p<0.001) than females. Male carriers for variant C exhibit a 10‐month delay in the onset of AD (p <0.05). One molecular function of PTPRS we chose to explore is macrophagy, given the implication of this biological process in the response against neurodegeneration. Macrophagy‐associated LC3 immunoreactivity occurs in most dystrophic neurites in AD and co‐localized with abnormal P‐tau in most neurofibrillary tangles. In the ROSMAP subjects, LC3 cortical protein levels were found to be significant reduced in C carriers

**Conclusions:**

PTPRS acts as a soluble, secreted neuroprotective receptor, found in the extracellular space in Alzheimer’s disease. These findings also highlight the functional importance of synaptic plasticity in the protection against neurodegenerative diseases.

